# Improved Code Team Performance and Outcomes After Implementation of Moderate Fidelity In Situ Simulation in a Pediatric Cardiac Acute Care Unit

**DOI:** 10.1007/s00246-024-03627-1

**Published:** 2024-08-21

**Authors:** Frances K. Woodard, Angela S. McKeta, Luke Schroeder, Sinai C. Zyblewski, Jason R. Buckley

**Affiliations:** Department of Pediatrics, Pediatric Nurse Practitioner, Medical University of South Carolina, 10 McClennan Banks Drive, Charleston, SC 29425 USA

**Keywords:** Simulation, Cardiac arrest, Pediatric, Cardiology

## Abstract

Children with cardiac disease suffer higher rates of in-hospital cardiac arrest. Cardiac arrest outside of the intensive care unit (ICU) is an infrequent event, which is a threat for suboptimal code team performance. Shorter time to the first epinephrine dose during cardiac arrest has been associated with improved survival and neurologic outcomes. Moderate fidelity in situ simulation training was implemented in the pediatric cardiac acute care unit (PCACU) to improve code team performance in August 2015. A small interprofessional team of simulation facilitators was developed that included nurses, physicians, and advanced practice providers. The primary outcome was time to first epinephrine dose in pulseless electrical activity (PEA) scenarios. Time to epinephrine of all simulation exercises, and actual cardiac arrests, that occurred in the PCACU were reviewed through May 2022. A total of 72 simulations were performed and 42 (58%) were PEA scenarios. A center line shift was observed for time to epinephrine for simulated PEA cardiac arrests (from 5 to 3 min). After implementation of simulation exercises, a center line shift was observed for time to epinephrine for actual cardiac arrests in the PCACU (from 8 to 2 min). Survival to hospital discharge after cardiac arrest improved after implementation of the training (0% vs. 64%, *p* = 0.02). Code team performance, as measured by time to epinephrine, can be improved following implementation of in situ simulation exercises in a pediatric cardiac acute care unit. Optimizing code team performance may contribute to improved patient outcomes.

**Supplementary Information:**

The online version contains supplementary material available at 10.1007/s00246-024-03627-1.

## Introduction

An estimated 15,000 children in the United States suffer cardiac arrest in the hospital each year [1]. Children with underlying cardiac disease are at increased risk of suffering in-hospital cardiac arrest (IHCA) but have a higher likelihood of survival after cardiac arrest compared to children without disease [2, 3].

Likelihood of survival with favorable neurological outcome can be improved if high-quality cardiopulmonary resuscitation (CPR) is initiated promptly. The American Heart Association (AHA) recommends that once recognition of a pulseless rhythm is identified, emergency response is activated immediately, high-quality chest compressions are started within 10 s, and epinephrine is administered within 5 min [4]. Delays in initiation of CPR and longer time to epinephrine administration have been associated with lower rates of return of spontaneous circulation (ROSC) and lower survival to hospital discharge [5, 6]. Similarly, timely defibrillation for shockable rhythms is more likely to result in ROSC [4, 7].

Basic Life Support (BLS) and Pediatric Advanced Life Support (PALS) are often required certifications for pediatric providers who may need to perform CPR. These courses require recertification every 2 years. Unfortunately, the skills and knowledge acquired during these certification courses may be lost quickly without routine practice [8]. The AHA has identified that only 14% of nurses retain advanced life support skills as far as one year out of certification [9]. In addition, training in a formal PALS or BLS classroom does not mirror the team composition or physical space of actual IHCA, which limits its capabilities as a training tool for these events.

The pediatric cardiac acute care unit (PCACU) at The Medical University of South Carolina (MUSC) is a 14-bed unit staffed by cardiac acute care nurses, pediatric cardiology fellows, pediatric residents, a physician assistant, and an attending pediatric cardiologist. Because of the increased rate of IHCA observed in children with underlying cardiac disease and the inadequacies of formal CPR training certifications, in situ simulation exercises were implemented in the PCACU in 2015 to improve code team performance. There is evidence that in situ simulation-based training can improve outcomes after pediatric IHCA, but improved outcomes outside of the ICU environment have not been reported [10]. The objective of this study is to describe the performance and impact of in situ simulation training in a pediatric cardiac acute care setting. The primary outcome was time to first epinephrine dose in both simulated and actual cardiac arrests.

## Materials and Methods

This study was approved by the Institutional Review Board at the Medical University of South Carolina. In 2015, an interprofessional team of simulation facilitators was created including nurses, physicians, and advanced practice providers. Simulation cases were developed based on actual patient events that occurred in the pediatric cardiac acute care unit and included the following:

1. Bradycardia/pulseless electrical activity (PEA) arrest in an infant with hypoplastic left heart syndrome;
2. Pulseless ventricular tachycardia/ventricular fibrillation arrest in an adolescent with cardiomyopathy;
3. Hypercyanotic spell in an infant with unrepaired tetralogy of Fallot; and
4. Supraventricular tachycardia in an infant recovering from cardiac surgery for transposition of the great arteries.

Case reference sheets were created for facilitator use (Electronic Supplementary Material 1). Room set-up, the simulation exercise, and the post-exercise debrief each lasted approximately 10 min. Since empty patient rooms and on-duty staff were utilized, the goal was to occupy no more than 30 min of room, or participant time.

Equipment purchased for the simulation exercises included a device that displays simulated patient vital sign data on real patient monitors (VitalsBridge^®^), two low-fidelity manikins (Laerdal Newborn Anne™ and Laerdal ECG Kid), and a Microsoft Surface Pro tablet. All additional equipment was acquired from existing resources including expired airway equipment, intravenous access catheters, and code medications.

Approximately two simulation exercises were scheduled every month and one of which was often canceled due to staff shortages or limited PCACU room availability. Exercises occurred on both day and night shifts. The participants included on-duty cardiac acute care nurses, pediatric residents, pediatric cardiology fellows, a clinical pharmacist (if available), and a respiratory therapist (if available). All team members in the PCACU were invited to participate but could opt out if active patient care responsibilities were necessary at the time. Approximately five nurses, two pediatric residents, and one pediatric cardiology fellow were expected to participate in each exercise. Based on nurse staffing data and the size of our pediatric residency and cardiology fellowship programs, this model would expose these team members (who are often the first responders to an emergency in this unit), to at least one simulation exercise per year. Data regarding the number of exercises performed by each individual participant (e.g., recurrent participation) were not collected. Prior to implementation, in situ simulation-based team training did not exist at our children’s hospital. Previous simulation experience of the participants (e.g., at a prior institution or in a simulation center) was unknown and not collected.

PCACU staff did not know the simulation exercise schedule (events were a surprise to the participants). Facilitator roles included the following: one person to control the simulated vital signs on the monitor (via the tablet), one person to describe the case scenario and address questions from the participants, and one person to record time of important events (e.g., time of epinephrine doses) using a timer and simulation exercise “scorecard” (Electronic Supplementary Material 2). Time to epinephrine administration or defibrillation (if indicated) was measured from when the manikin was “made” pulseless by the facilitators.

The post-event “hot” debrief duration was approximately 10 min and followed a standard format that aligned with a hospital-wide post-event debriefing tool. This tool applies similar methodology as the plus-delta approach [11]. After a period of participant self-reflection about what did and did not go well during the exercise, the facilitator team provided performance data (e.g., time to epinephrine or defibrillation) and case-specific feedback about improvement strategies. Later that day or the following day, a post-event “cold” debrief involved an email to all participants with the team’s “Mock Code Scorecard” attached (Electronic Supplemental Material 2).

An internal database of actual cardiac arrests that occurred in the PCACU from 2011 to 2022 was used to compare the time to the first epinephrine dose and patient outcomes before and after implementation of in situ simulation exercises. Bivariate comparisons of patients who suffered cardiac arrest before and after implementation of in situ simulation exercises were performed using Fisher’s exact or Mann–Whitney U tests as appropriate for individual variables. All statistical analyses were performed using SPSS version 28 (IBM, Armonk, NY).

## Results

Seventy-two in situ simulation exercises were performed in the PCACU from August 2015 to May 2022. Table 1 summarizes the exercises that were performed during the study period and the estimated number of participants. Forty-two (58%) of the exercises were simulated pulseless electrical activity (PEA) cardiac arrests and 25 (35%) were simulated pulseless ventricular tachycardia or ventricular fibrillation (pVT/VF) cardiac arrests. The five remaining cases simulated unstable supraventricular tachycardia or hypercyanotic spells associated with tetralogy of Fallot; these cases did not result in simulated cardiac arrest and thus are not included in the analysis. Based on the estimated number of participants, nurse staffing data, pediatric residency and cardiology fellowship training program sizes, the exposure rate to simulation exercises was as follows: two exercises per year for each PCACU nurse, one exercise per year for each pediatric resident, and one exercise per year for each pediatric cardiology fellow.

**Table 1:** In situ simulation exercises performed in the PCACU, 2015–2022

Variable	PEA, *n* = 42	VT/VF, *n* = 25	Other, *n* = 5	Total
Academic year	9		1	
2016		5		15
2017	13	11	3	27
2018	3	1	0	4
2019	4	3	1	8
2020	3	2	0	5
2021	6	3	0	9
2022	4	0	0	4
Exercises per year, median	4	3	0	8
Day shift	30	13	2	45
Night shift	12	12	3	27
Participants (estimates)				
Nurses	210	125	25	360
Pediatric residents	84	50	10	144
Pediatric cardiology fellows	42	25	5	72

PEA, pulseless electrical activity; VT/VF, ventricular tachycardia, or ventricular fibrillation

**Fig. 1:**
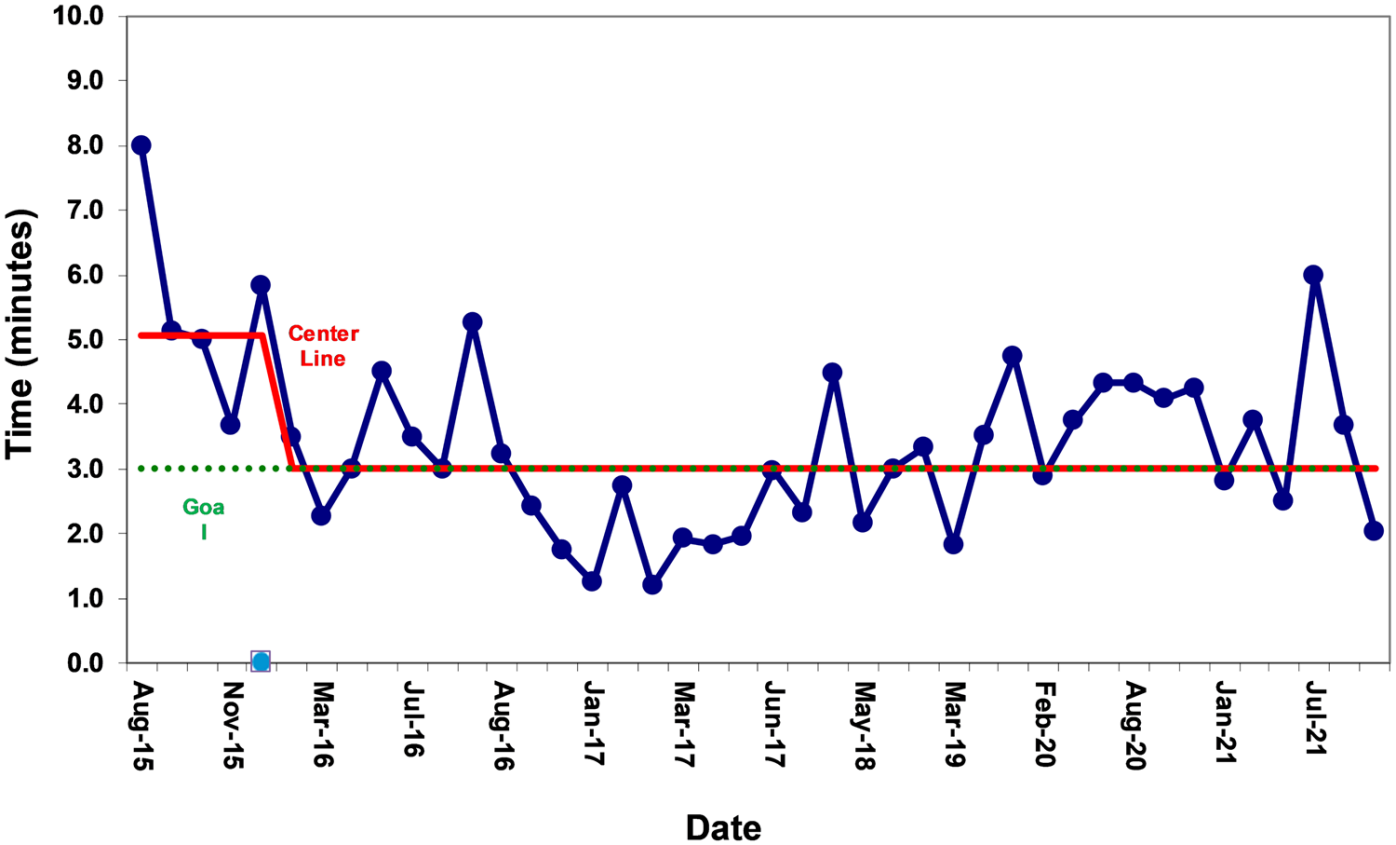
Run chart of time to first dose of epinephrine for in situ simulation pulseless electrical activity (PEA) exercises. A center line (red line) shift was observed demonstrating a decrease in time to epinephrine during the exercises from 5 to 3 min

Of the 42 PEA exercises, 30 (71%) were facilitated during the day shift (7 AM–7 PM) and 12 (29%) occurred during the night shift. A median of four PEA arrest exercises were performed per year. A center line shift was observed for time to epinephrine during PEA exercises from a median of 5–3 min during the study period (Fig. 1).

**Fig. 2:**
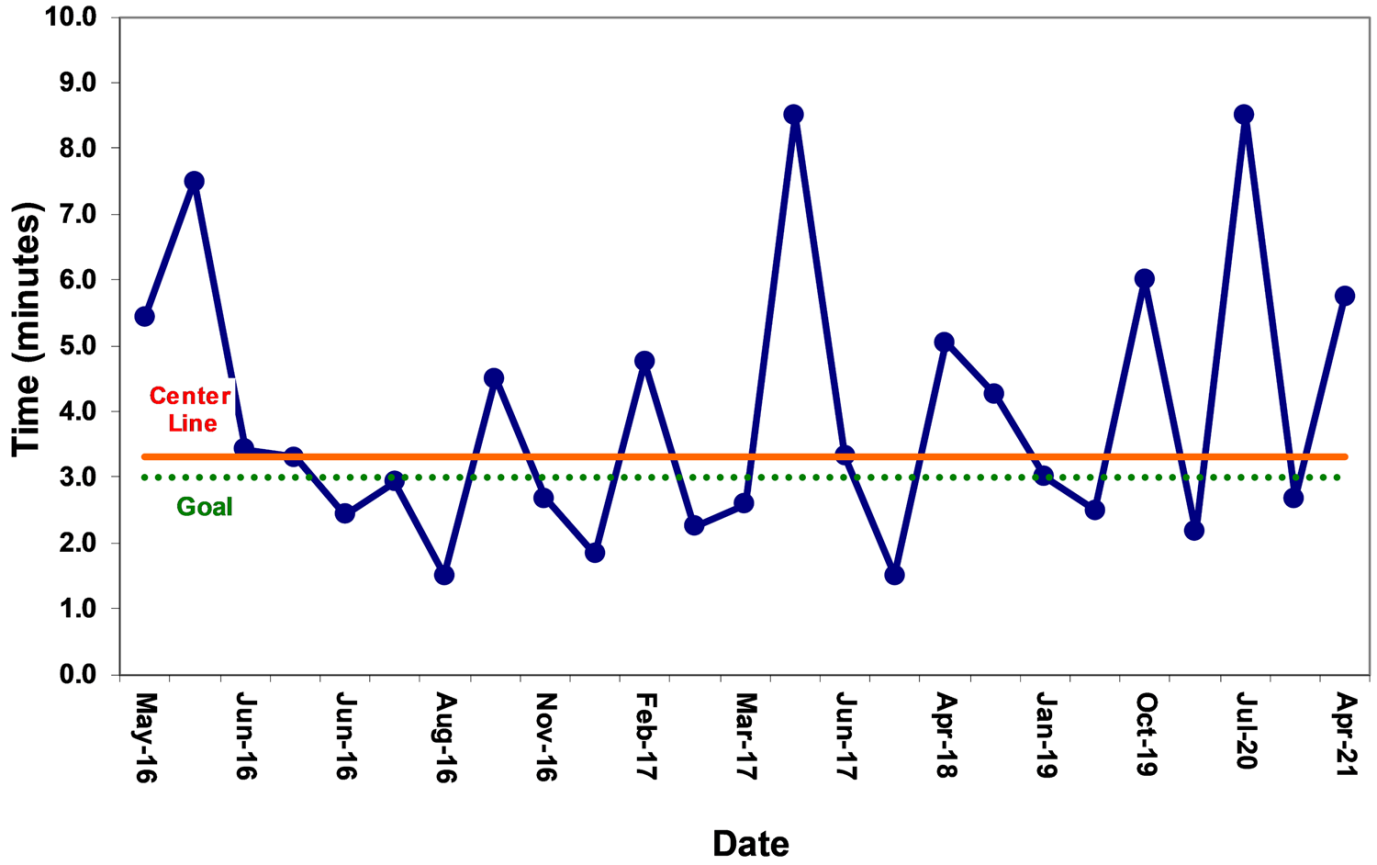
Run chart of time to defibrillation for in situ simulation ventricular tachycardia or ventricular fibrillation (VT/VF) exercises. No change in the center line (red line) was observed

Of the 25 pVT/VF exercises, 13 (52%) were facilitated occurring during the day shift and 12 (48%) occurred during the night shift. A median of three pVT/VF arrest exercises were performed per academic year. Median time to defibrillation was 3.3 min and did not change during the study period (Fig. 2).

**Fig. 3:**
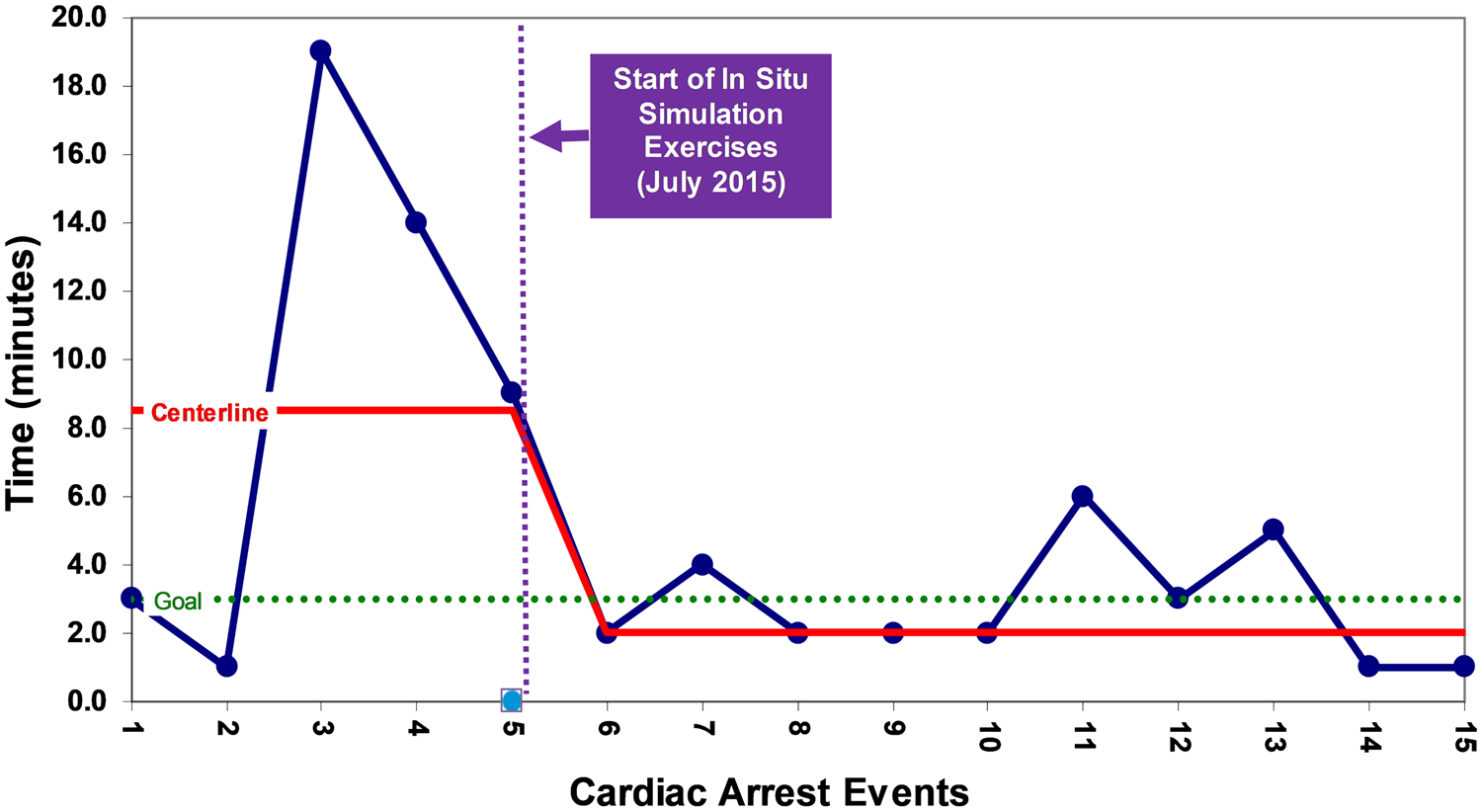
Run chart of time to first dose of epinephrine for actual cardiac arrest events that occurred in the pediatric cardiac acute care unit (PCACU) during the study period. A center line (red line) shift was observed after implementation of in situ simulation exercises demonstrating a decrease in time to epinephrine during the events from 8 to 2 min

Table 2 summarizes actual cardiac arrests that have occurred in the PCACU. Prior to implementation of in situ simulation exercises, five patients suffered cardiac arrest from 2011 to 2015. The median time to epinephrine for these events was 8.5 min and zero patients survived the arrest. Since the implementation of simulation training, there have been 14 cardiac arrests in the PCACU from 2015 to 2023. Eleven (79%) of these patients received epinephrine during the resuscitation. The median time to epinephrine for these events was 2.0 min (Fig. 3). Twelve (86%) of the fourteen patients achieved ROSC, and the other two patients underwent successful extracorporeal cardiopulmonary resuscitation (ECPR). Nine (64%) of the fourteen patients survived to hospital discharge, including both patients who received ECPR.

**Table 2:** Actual cardiac arrests in the PCACU, 2011–2023

	Pre-simulation training, *n* = 5	Post-simulation training, *n* = 14	*p*-value
Time to epinephrine, minutes	8.5	2.0	0.13
ROSC	0 (0%)	12 (86%)	< 0.01
ECPR	0 (0%)	2 (14%)	0.53
Survived to hospital discharge	0 (0%)	9 (64%)	0.02

ROSC, return of spontaneous circulation; ECPR, extracorporeal cardiopulmonary resuscitation

## Discussion

To our knowledge, this is the first report describing implementation of an in situ “mock code” simulation program in a pediatric cardiac acute care unit. Improved code team performance as measured by decreased time to epinephrine during simulation exercises, and actual cardiac arrests, was observed over time. Since implementation of the program in 2015, ROSC and survival to hospital discharge after cardiac arrest in the PCACU have improved significantly. Because IHCA occurs more frequently in children with cardiac disease, and because mock code exercises are resource-intensive, the PCACU may represent a high yield setting (outside of the ICU) to focus in situ simulation training initiatives.

Previous studies describing in situ simulation training in children’s hospitals and its impact on CPR performance and IHCA patient outcomes have demonstrated mixed results [10, 12, 13]. These studies performed simulation exercises across multiple units throughout their respective hospitals. Andreatta et al. reported improved survival after IHCA following implementation of a hospital-wide, simulation-based mock code program. Importantly, the location of IHCA events was not reported in this study and thus it is not clear if improved survival was observed in non-ICU environments. Over 90% of pediatric IHCA occurs in an ICU, which in some ways makes it an easier environment to improve CPR performance and outcomes [14]. Moreover, facilitating and debriefing in situ simulation exercises is resource-intensive, and a large-scale effort across the entire hospital may not be feasible, or sustainable, at some centers. Ensuring that every nurse and physician is exposed to training would require frequent exercises in all units and large participant teams. Increasingly, hospitalized children with cardiac disease are being cared for in separate cardiac units with dedicated care teams [15–17]. Because of the inherent risk of this patient population, and the resource-intensive nature of the intervention, the pediatric cardiac acute care unit may be a valuable location for a more targeted approach to implementing a mock code program.

Time to the first dose of epinephrine during cardiac arrest has been associated with ROSC, neurologic outcomes, and survival to hospital discharge after pediatric IHCA in two large studies that utilized the Get With The Guidelines-Resuscitation (GWTHG-R) registry [5, 6]. Timely administration of epinephrine is unlikely to be helpful unless high-quality CPR, effective code team leadership, and communication are performed [12]. Thus, the findings of Andersen and Raymond et al. suggest that time to epinephrine may represent a useful, objective, surrogate metric of overall code team performance.

This study has multiple limitations. Due to the small number of actual cardiac arrests and the potential for unmeasured confounders, we have been careful to designate the described improvements as observations rather than associations. Concurrent process improvement activities were implemented during the study period which could confound the findings. An early warning score and a dedicated rapid response team were developed and implemented in 2015. It is possible that these tools have allowed for earlier identification of patient deterioration in the PCACU, timelier interventions, and improved patient outcomes. However, these tools were developed to improve surveillance and prevent cardiac arrest, whereas in situ simulation training has focused on code team performance. Additionally, patient volume or acuity has not declined in the PCACU during the study period. To the contrary, patients on this unit have increasingly received therapies that are often associated with higher illness severity and include high flow nasal cannula, inotropic medications (e.g., milrinone, dopamine), prostaglandin E infusions, and ventricular assist devices. Increasing severity of illness in this unit may have inherently increased the proficiency of the care team over time, improved resuscitations, and confounded the findings. Lastly, the structure of our hospital code team has not changed significantly during the study period, but the utilization of ECPR has become more prevalent.

In conclusion, we have observed improved team performance during in situ simulation exercises and improved patient outcomes after cardiac arrest in the PCACU. We recommend focusing simulation training resources to this high-risk hospitalized patient population and using time to epinephrine as a surrogate outcome measure of code team performance.

## Supplementary Information

Below is the link to the electronic supplementary material.

Supplementary file 1 (PDF 124 kb)

Supplementary file 2 (PDF 109 kb)

## Data Availability

No datasets were generated or analysed during the current study.

## References

[1] Holmberg M, Wiberg S, Ross C, Kleinman M, Hoeyer-Nielsen A, Donnino M et al (2019) Trends in survival after pediatric in-hospital cardiac arrest in the United States. Circulation 140:1398–1408. 10.1161/CIRCULATIONAHA.119.041667 10.1161/CIRCULATIONAHA.119.041667 PMC6803102 31542952

[2] Ortmann L, Prodhan P, Gosset J, Schnayder S, Berg R, Nadkarni V et al (2011) Outcomes after in-hospital arrest in children with cardiac disease. Circulation 124:2329–2337. 10.1161/CIRCULATIONAHA.110.01346 22025603 10.1161/CIRCULATIONAHA.110.013466

[3] Mir T, Shafi O, Uddin M, Nadiger M, Llah F, Qureshi W et al (2022) Pediatric cardiac arrest outcomes in the United States: a nationwide database cohort study. Cureus. 10.7759/cureus.26505 10.7759/cureus.26505 PMC9339595 35923483

[4] Topjian AA, Raymond, TT, Atkins D, Chan M, Duff JP, Joyner BL et al (2020) Part 4: Pediatric basic and advanced life support 2020 American Heart Association guidelines for cardiopulmonary resuscitation and emergency cardiovascular care. Circulation 142:S469–S523. 10.1161/CIR.0000000000000901 10.1161/CIR.0000000000000901 33081526

[5] Raymond T, Praestgaard A, Berg R, Nadkarni V, Parshuram C (2019) The association of hospital rate of delayed epinephrine administration with survival to discharge for pediatric nonshockable in-hospital cardiac arrest. Pediatr Crit Care Med 20:405–416. 10.1097/PCC.0000000000001863 10.1097/PCC.0000000000001863 30672841

[6] Anderson L, Berg K, Saindon B, Massaro J, Raymond T, Berg R et al (2015) Time to epinephrine and survival after pediatric in-hospital cardiac arrest. JAMA 314(8):802–810 10.1001/jama.2015.9678 10.1001/jama.2015.9678 PMC6191294 26305650

[7] Topjian AA (2018) Shorter time to defibrillation in pediatric CPR: children are not small adults, but shock them like they are. JAMA Netw Open. 1(5):e182653. 10.1001/jamanetworkopen.2018.2653 10.1001/jamanetworkopen.2018.2653 30646162

[8] Herbers M, Heaser J (2016) Implementing an in situ mock code quality improvement program. Am J Crit Care 25:393–399. 10.4037/ajcc2016583 27587418 10.4037/ajcc2016583

[9] Hammontree J, Kinderknecht C (2022) An in situ mock code program in the pediatric intensive care unit: a multimodal nurse-led quality improvement initiative. Crit Care Nurse 42:42–55. 10.4037/ccn2022631 35362083 10.4037/ccn2022631

[10] Andreatta P, Saxton E, Thompson M, Annich G (2011) Simulation-based mock codes significantly correlate with improved pediatric patient cardiopulmonary arrest survival rates. Pediatr Crit Care Med 12(1):33–38. 10.1097/PCC.0b013e3181e89270 10.1097/PCC.0b013e3181e89270 20581734

[11] Cheng A, Eppich W, Epps C, Kolbe M, Meguerdichian M, Grant V (2021) Embracing informed learner self-assessment during debriefing: the art of plus-delta. Adv Simul (Lond) 6(1):22. 10.1186/s41077-021-00173-1 10.1186/s41077-021-00173-1 PMC8180042 34090514

[12] Clarke S, Julie I, Yao A, Bang H, Barton J, Alsomali S et al (2019) Longitudinal exploration of in situ mock code events and performance of cardiac arrest skills. BMJ Simul Technol Enhanc Learn. 5:22–26. 10.1136/bmjstel-2017-000255 10.1136/bmjstel-2017-000255 PMC6289268 30555719

[13] Wise K, Zinkan L, Rutledge C, Gaither S, Norwood C, Tofil N (2020) Development of a “First five minutes” program to improve staff response to pediatric codes. Am J Crit Care 29:233–236. 10.4037/ajcc2020407 32355972 10.4037/ajcc2020407

[14] Berg RA, Sutton RM, Holubkov R, Nicholson CE, Dean JM, Harrison R et al (2013) Ratio of PICU versus ward cardiopulmonary resuscitation events is increasing. Crit Care Med 10:2292–7. 10.1097/CCM.0b013e31828cf0c0 10.1097/CCM.0b013e31828cf0c0 PMC3783604 23921270

[15] Burstein DS, Jacobs JP, Li JS, Sheng S, O’Brien SM, Rossi AF et al (2011) Care models and associated outcomes in congenital heart surgery. Pediatrics 6:e1482–9. 10.1542/peds.2010-2796 10.1542/peds.2010-2796 PMC3103274 21576309

[16] Burstein DS, Rossi AF, Jacobs JP, Checchia PA, Wernovsky G, Li JS, Pasquali SK (2010) Variation in models of care delivery for children undergoing congenital heart surgery in the United States. World J Pediatr Congenit Heart Surg 1:8–14. 10.1177/2150135109360915 10.1177/2150135109360915 PMC3285260 22368780

[17] Kipps AK, Cassidy SC, Strohacker CM, Graupe M, Bates KE, McLellan MC et al (2018) Collective quality improvement in the paediatric cardiology acute care unit: establishment of the Pediatric Acute Care Cardiology Collaborative (PAC3). Cardiol Young 8:1019–1023. 10.1017/S1047951118000811 10.1017/S1047951118000811 29952278

