# Supplementary material for: Improved Code Team Performance and Outcomes After Implementation of Moderate Fidelity In Situ Simulation in a Pediatric Cardiac Acute Care Unit
Source: Pediatr Cardiol. 2024 Aug 21;46(8):2230–5. doi: 10.1007/s00246-024-03627-1 (PMC12583419; doi:10.1007/s00246-024-03627-1)
Supplement: Supplementary file 1 — (PDF 124 kb) [file 246_2024_3627_MOESM1_ESM.pdf]

**Mock Code Scorecard**

| **Stage 1: First 5 minutes – Recognition, CPR start, CODE Activation** | | |
|---|---|---|
| **Critical Step** | **Score** | **Comments** |
| **Recognition/CPR initiation** | | ○ Pulse checked<br>○ CPR started |
| **Code called/cart to bedside** | | ○ Code button pressed<br>○ Confirmation code has been called<br>○ Code cart brought to room < 90 seconds |
| **High quality CPR performed** | | ○ Compression rate 100-120/min<br>○ Backboard used<br>○ 15:2 compression/ventilation ratio used |
| **Roles clearly assigned** | | ○ Roles assigned (airway, compressions, recorder, meds/monitor) |
| **Meds administered and/or defibrillation performed** | | ○ Epi (code dose) given < 3 minutes<br>○ Defibrillation (2J/kg) < 3 minutes |
| **Stage 2: Next 10 minutes – Code Team Performance** | | |
| **Critical Step** | **Score** | **Comments** |
| **Team leader initial survey and leadership** | | ○ Event history obtained<br>○ Confirmation code called<br>○ Assessed IV access status<br>○ Ensured high-quality CPR is in progress<br>○ Roles assigned |
| **High quality CPR performed** | | ○ Compression rate 100-120<br>○ Backboard used<br>○ 15:2 compression/ventilation ratio<br>○ Pulse checks q2 minutes w/compressor switches<br>○ Rhythm assessed **and verbalized** during pulse checks |
| **IV/IO access obtained or confirmed** | | ○ IV access confirmed |
| **Intubation and/or post-ROSC care** | | ○ Airway established<br>○ Check BP<br>○ Blood gas/labs, CXR after ROSC |
| **Communication and teamwork** | | ○ Clear messages from code leader<br>○ Closed loop communication<br>○ Active code recorder |
| **Total Score** | | |

**<u>Data Summary</u>:**

| | |
|---|---|
| **Mock Code Overall Score** | |
| **Recognition of arrest** | |
| **Initiation of CPR** | |
| **Time to Epi** | |
| **Time to defibrillation** | |
