# Supplementary material for: Improved Code Team Performance and Outcomes After Implementation of Moderate Fidelity In Situ Simulation in a Pediatric Cardiac Acute Care Unit
Source: Pediatr Cardiol. 2024 Aug 21;46(8):2230–5. doi: 10.1007/s00246-024-03627-1 (PMC12583419; doi:10.1007/s00246-024-03627-1)
Supplement: Supplementary file 2 — (PDF 109 kb) [file 246_2024_3627_MOESM2_ESM.pdf]

Case 1: Bradycardia / PEA arrest in infant with HLHS

Patient information/Case stem

You are called to evaluate a 3-week-old infant with Hypoplastic Left Heart Syndrome (HLHS) status post Norwood procedure who is currently on the ward "working on feeds". The nurse informs you that he just vomited for the 3rd time today, is "breathing harder" and looks pale. He is irritable and inconsolable.

Initial Monitoring Parameters (vitals)

Weight: 4 kg
Temp: 35.3 degrees Celsius
HR: 130
BP: 50/22
RR: 40
SpO2: 73%

General Case Outline

☐ Use mock code scorecard to record observations and measurements
☐ Initial assessment by participant – audible shunt murmur (if asked)
☐ Patient HR and O2 saturation decrease
☐ Loss of pulse, loss of O2 saturation waveform
☐ Monitor rhythm = bradycardia / PEA
☐ ROSC after at least 3 rounds of CPR and 2 doses of epinephrine
☐ Consider delayed ROSC if low-quality CPR
☐ Post-event debrief with reflective learning and team performance data

Case 2: Pulseless VT / VF arrest in adolescent with cardiomyopathy

Patient information/Case stem
You are called to evaluate a 12-year-old for a change in rhythm on the monitor. The patient was admitted for evaluation and management of a new diagnosis of cardiomyopathy and was having PVCs last night. The patient appears pale and is disoriented.

Initial Monitoring Parameters (vitals)
Weight: 50 kg
Temp: 37 degrees Celsius
HR: 170
BP: 60 / 30
RR: 20
SpO2: 86%

General Case Outline
☐ Use mock code scorecard to record observations and measurements
☐ Initial assessment by participant – pale, altered mental status, questionable/thready pulse
☐ Loss of pulse, loss of O2 saturation waveform, loss of consciousness (if asked)
☐ Monitor rhythm = pulseless VT
☐ ROSC after at least 3 rounds of CPR and 2 defibrillations
☐ Consider delayed ROSC with change in rhythm to VF if low-quality resuscitation
☐ Post-event debrief with reflective learning and team performance data

Case 3: Hypercyanotic Event in infant with Tetralogy of Fallot (TOF)

Patient information/Case stem
You are called to supervise IV placement on a 4-month-old infant with TOF admitted for overnight hydration prior to surgery tomorrow. The infant has severe multi-level pulmonary stenosis. The nurse gets the IV but toward the end of the procedure, and as the oxygen saturation monitor starts to pick up again, you note that oxygen saturations are significantly decreased (baseline is 88%) and the patient appears cyanotic.

Vital signs: HR 160s, BP: unable to obtain, oxygen saturations: 50s

Initial Monitoring Parameters (vitals)
Weight: 7 kg
Temp: 36.8 degrees Celsius
HR: 110
BP: 95/40
RR: 30s
SpO2: 83%
Last feed: 3 hours ago

General Case Outline
☐ Use mock code scorecard to record observations and measurements
☐ Initial assessment by participant – no murmur present (if asked)
☐ Patient HR increases and O2 saturation decreases (HR = 160s, O2 sat = 60s)
☐ Patient maintains a pulse and consciousness (if asked)
☐ Monitor rhythm = sinus tachycardia
☐ Improved oxygen saturation after minimum of 3 strategies utilized
  - ☐ Blow-by oxygen
  - ☐ Knee to chest position
  - ☐ Fluid bolus
  - ☐ Sedation (e.g. IV/IM morphine)

☐ Post-event debrief with reflective learning and discussion of advanced therapies and interventions for hypercyanotic spells

Case 4: Supraventricular Tachycardia (SVT)

Patient information/Case stem
You are called to evaluate a 1 month with transposition of the great arteries status post arterial switch operation. The telemetry tech reports that the patient has been tachycardic for the past few minutes. By history, the patient has frequent atrial ectopy in the ICU but was not started on any medications for this issue. Echocardiogram yesterday demonstrated mildly decreased biventricular function and no effusion.

Vital signs: HR 240, BP: 90/50, oxygen saturations: high 90s, RR 40s

Initial Monitoring Parameters (vitals)
Weight: 5kg
Temp: 36.8 degrees Celsius
HR: as high as 240 but has had some HRs in the 120s
BP: 75/40
RR: 30s
SpO2: 100%
Last feed: 3 hours ago

General Case Outline
☐ Use mock code scorecard to record observations and measurements
☐ Initial assessment by participant – patient crying and well perfused (if asked)
☐ Monitor rhythm (or EKG if asked for) = SVT
☐ No improvement in rhythm despite initial strategies utilized:
  ☐ Vagal maneuvers
  ☐ Adenosine
  ☐ Rapid atrial pacing (if asked for by participants)
☐ Subsequent BP on monitor decreases after initial therapies (e.g. BP = 48/26)
☐ Patient appears pale, is grunting, 1+ pulses (if asked)
☐ Rhythm converts to sinus rhythm and vital signs improve if proper synchronized cardioversion performed
☐ Post-event debrief with reflective learning and discussion of advanced therapies and interventions for SVT
